# Turning alterations detected by mobile health technology in idiopathic REM sleep behavior disorder

**DOI:** 10.1038/s41531-024-00682-6

**Published:** 2024-03-18

**Authors:** Cinzia Zatti, Andrea Pilotto, Clint Hansen, Andrea Rizzardi, Marcello Catania, Robbin Romijnders, Leandro Purin, Maria P. Pasolini, Eva Schaeffer, Andrea Galbiati, Luigi Ferini-Strambi, Daniela Berg, Walter Maetzler, Alessandro Padovani

**Affiliations:** 1 Department of Clinical and Experimental Sciences, Neurology Unit, University of Brescia, Brescia, Italy; 2 Laboratory of digital Neurology and biosensors, University of Brescia, Brescia, Italy; 3 Department of continuity of care and frailty, Neurology Unit, ASST Spedali Civili of Brescia, Brescia, Italy; 4 Department of Neurology, University Hospital Schleswig-Holstein and Kiel University, Kiel, Germany; 5 Department of Clinical and Experimental Sciences, Neurophysiology Unit, University of Brescia, Brescia, Italy; 6 IRCCS San Raffaele Scientific Institute, Department of Clinical Neurosciences, Neurology-Sleep Disorders Centre, Milan, Italy; 7 Faculty of Psychology, “Vita-Salute” San Raffaele University, Milan, Italy

**Keywords:** Research data, Epidemiology

## Abstract

Idiopathic REM sleep Behavior Disorder (iRBD) is a condition at high risk of developing Parkinson’s disease (PD) and other alpha-synucleinopathies. The aim of the study was to evaluate subtle turning alterations by using Mobile health technology in iRBD individuals without subthreshold parkinsonism. A total of 148 participants (23 persons with polysomnography-confirmed iRBD without subthreshold parkinsonism, 60 drug-naïve PD patients, and 65 age-matched controls were included in this prospective cross-sectional study. All underwent a multidimensional assessment including cognitive and non-motor symptoms assessment. Then a Timed-Up-and-Go test (TUG) at normal and fast speed was performed using mobile health technology on the lower back (Rehagait®, Hasomed, Germany). Duration, mean, and peak angular velocities of the turns were compared using a multivariate model correcting for age and sex. Compared to controls, PD patients showed longer turn durations and lower mean and peak angular velocities of the turns in both TUGs (all *p* ≤ 0.001). iRBD participants also showed a longer turn duration and lower mean (*p* = 0.006) and peak angular velocities (*p* < 0.001) compared to controls, but only in the TUG at normal speed. Mobile health technology assessment identified subtle alterations of turning in subjects with iRBD in usual, but not fast speed. Longitudinal studies are warranted to evaluate the value of objective turning parameters in defining the risk of conversion to PD in iRBD and in tracking motor progression in prodromal PD.

## Introduction

Idiopathic Rapid Eye Movement sleep behavior disorder (iRBD) is the diagnosis associated with the highest risk of developing an alpha-synucleinopathy, particularly Parkinson’s disease (PD) and dementia with Lewy bodies (DLB)^1^. The overall risk of conversion within 10–12 years is 60–80%^1^. Clinically, iRBD is defined by abnormal dream enacting behaviors with a persisting of muscle tone during the REM phases of sleep, in which the muscle tone is normally markedly reduced. The gold standard for the diagnosis and evaluation of iRBD is video-polysomnography (PSG)^2^.

Therefore, iRBD represents an important model to study disease progression in prodromal phases, when no or only subtle motor features are present^3^. It was already shown that iRBD can be associated with mild Parkinsonian signs, such as isolated upper-limb bradykinesia, tremor, and reduced arm-swing. However, to our best knowledge, no study to date investigated turning performance in prodromal PD phases. We therefore aimed at evaluating turning in persons with iRBD using mobile health technology.

## Results

Twenty five consecutive iRBD patients, 75 age-matched drug-naïve patients with parkinsonism, and 65 age-matched controls were screened. 2 iRBD were excluded due to the presence of comorbid obstructive apnoea and alcohol abuse. From the group of drug-naïve parkinsonism, 15 participants were excluded due to signs for atypical parkinsonism (*n* = 4), isolated tremor (*n* = 8), drug-induced parkinsonism (*n* = 2) and severe leukoencephalopathy at MRI (*n* = 1).

The demographic characteristics and clinical features of the participants are shown in Table 1. PD and iRBD showed similar prevalence and severity of non-motor features. As expected by applying the exclusion criteria, none of the iRBD patients exhibited subthreshold parkinsonism (mean 2 ± 2 points, non-significant to controls), as defined by prodromal MDS criteria, that is an MDS-UPDRS-III score >6 excluding postural and action tremor^4^. No iRBD participant exhibited cognitive impairment.

**Table 1:** Demographics and clinical characteristics in controls, iRBD and PD

	CP	iRBD	PD	*P*-value*
Participants [n]	65	23	60	
Age [y]	69 ± 6	72 ± 6	68 ± 8	0.055 ^a^
Height [m]	1.7 ± 0.1	1.7 ± 0.1	1.7 ± 0.1	0.340^a^
Sex M/F (%M)	26/39(40)	19/4(83)	32/28(53)	**0.002**^**b**^ *#
Disease Duration [y]	-	-	1 ± 1	
RBD symptoms duration		4 ± 3		
MoCA(0-30) [pts]	27 ± 2	25 ± 3	24 ± 3	**<0.001**^**c**^ **§**
MDS-UPDRS-I(0-52) [pts]	1 ± 12	6 ± 5	5 ± 5	**<0.001**^**c**^ *§
MDS-UPDRS-II(0-52) [pts]	0 ± 1	1 ± 1	4 ± 4	**<0.001**^**c**^ **§#**
MDS-UPDRS-III (0-132) [pts]	2 ± 3	2 ± 2	16 ± 10	**<0.001**^**c**^ **§#**
RBDSQ(0-13) [pts]	0 ± 1	9 ± 3	3 ± 3	**<0.001**^**c**^ *#§
NMSS (0-360) [pts]	1 ± 1	22 ± 17	21 ± 21	**<0.001**^**c**^ *§
Sniffing Sticks(0-12) [pts]	9 ± 2	5 ± 3	5 ± 2	**<0.001**^**c**^ *§
Hyposmic subjects [n] (%)	4 (6%)	14 (61%) (60.8%)	45 (75%)	**<0.001**^**c**^ *§

Pairwise with Bonferroni Correction for three comparisons (*iRBD vs CP; ^§^PD vs CP; ^#^ iRBD vs PD). *p*-value < 0.05 was considered as statistically significant.

^a^Parametric ANOVA.

^b^Chi Square test.

^c^Non-parametric comparison corrected for age and sex.

*BDI* Beck Depression Inventory, *CP* control participants, *iRBD* subjects with idiopatic REM sleep behavior disorders, *MDS-UPDRS* Movement Disorder Society Unified Parkinson’s disease Rating scale, *MoCA* Montreal Cognitive Assessment, *NPI* neuropsychiatric inventory, *NMSS* non motor symptom scale, *PD* Parkinson’s disease patients, *RBDSQ* RBD screening questionnaire.

### Mobile Health technology timed up and go assessment

Table 2 shows the total time of TUG and the digital parameters related to turning in the three groups. The overall duration of TUG tests was longer in PD compared to controls, with no differences between RBD and controls at normal and fast speed.

**Table 2:** TUG parameters in controls, iRBD and PD

	CP (65)	iRBD (23)	PD (60)	*P*-value*	iRBD-CP difference	PD-CP difference	PD-iRBD difference
*TUG NORMAL SPEED*							
TUG duration (s)	11.3 ± 2.09	12.8 ± 1.8	14.0 ± 3.5	**<0.001**^**b**^ **§**	1.4 ± 0.8	2.4±0.5	1 ± 0.8
Duration Of Turns (s)	2.3 ± 0.4	2.8 ± 0.4	3.0 ± 0.6	**<0.001**^**a**^ **§**	0.5 ± 0.1	0.7±0.1	0.2 ± 0.1
Mean Angular Velocity (°/s)	82 ± 14	67 ± 8	61 ± 15	**<0.001**^**a**^ *§	−15.0 ± 3.4	−20.4±2.4	−5.4 ± 3
Peak Angular Velocity (°/s)	194 ± 34	152 ± 22	138 ± 28	**<0.001**^**a**^ *§	−41.9 ± 7.7	−54.0±5.5	−12.1 ± 7.5
*TUG FAST SPEED*							
TUG duration (s)	8.9 ± 1.8	9.2 ± 0.9	10.9 ± 2.4	**<0.001**^**b**^ **§#**	0.3 ± 0.6	1.8±0.4	1.5 ± 0.5
% Variation TUG duration	−21.8 ± 8.8	-27.7 ± 6.4	-20.3 ± 6.7	**0.011**^**b**^ *#			
Duration Of Turns (s)	1.9 ± 0.3	2.2 ± 0.4	2.5 ± 0.5	**<0.001**^**b**^ **§#**	0.3 ± 0.1	0.6±0.1	0.3 ± 0.1
% Variation Turning duration	−16.6 ± 12.6	−21.22 ± 12.3	−13.8 ± 10.2	0.042^a^			
Mean Angular Velocity (°/s)	98 ± 18	85 ± 15	72 ± 16	**<0.001**^**a**^ **§#**	−12.9 ± 4.2	−24.4±2.9	−11.5 ± 4.1
%VariationMeanVelocity	20.9 ± 16.4	26.1 ± 18.4	16.8 ± 13.2	0.219^b^			
Peak Angular Velocity (°/s)	235 ± 40	203 ± 37	172 ± 35	**<0.001**^**a**^ **§#**	−32 ± 9.4	−62.8 ± 6.7	−30.8 ± 9.2
%VariationPeakVelocity	22.6 ± 13.2	30.9 ± 20.3	22.9 ± 14.8	0.822^b^			

Pairwise with Bonferroni Correction for 3 comparisons (*iRBD vs CP; ^§^PD vs CP; ^#^iRBD vs PD). *p*-value < 0.006 was considered as statistically significant for digital extracted variables.

^a^Parametric comparison corrected for age and sex

^b^Non-parametric comparison corrected for age and sex

*CP* control participants, *iRBD* subjects with idiopatic REM sleep behavior disorders, *PD* Parkinson’s disease patients, *TUG* timed up and go test.

In both normal and fast speed assessments, drug-naïve PD but not iRBD showed significantly longer turning duration and slower turning speed compared to controls. The digital analyses on normal speed turning revealed lower mean and peak angular velocity in iRBD compared to controls (respectively *p* = 0.006 and *p* < 0.001 with absolute differences of −15.0 ± 3.4 and −41.9 ± 7.7). Compared to PD, iRBD showed a similar duration of turns but higher mean and peak angular velocity.

At fast speed, PD showed longer turning duration and lower angular velocity (peak and mean) compared to controls (*p* < 0.001 with absolute differences of 0.7 ± 0.1, −54.0 ± 5.5, and −20.4 ± 2.4, respectively). Conversely, iRBD did not differ significantly from controls in turning duration, mean, and maximum velocities.

The variation percentages of TUG duration were significantly greater in iRBD in comparison to controls and PD; a similar (not significant) trend of percentage of variation was observed in iRBD for mean and peak angular velocities- which resulted higher in this group compared to PD and controls.

The analysis of clockwise and counter-clockwise tests separated are present as supplementary material and showed a similar pattern of distribution from PD, iRBD and PD—with iRBD vs controls differences more evident for left vs right turning (Supplementary Table 1). Clockwise and counter-clockwise parameters in people with Parkinson’s disease (PD) in which the left side is most affected (PD Left side) and in which the right side is most affected (PD Right side) did not show significative differences (Supplementary Table 2).

## Discussion

The group of iRBD is at high risk of conversion to alpha-synucleinopathy and is thus target of several studies focusing on markers to track motor progression over time^3,5,6^ (Supplementary Table 3). Still, the relevance of subtle motor impairment and its progression over time in prodromal PD stages are still not well understood. Findings showed that the standard measurement of time for completing the TUG test were able to differentiate PD but not iRBD from matched controls. Conversely, the digital assessment of turning detected important differences between iRBD and controls, even in the absence of any relevant Parkinsonian signs at normal examination. Our analyses were indeed able to detect significant subclinical differences between groups, below the minimal clinically significant value for the time duration of TUG, set at 3.5 s^7^. The digital assessment analyses were focused on turning duration, mean, and peak angular velocity, which are standard parameters already demonstrated to differentiate PD from controls in different cohorts^8^. Turning performances were evaluated in TUG performed separately at normal and fast speeds, as tasks performed at higher speeds might be more sensitive compared to the usual speed.

In TUG performed at normal speed, iRBDs exhibited increased duration of turning and decreased mean and peak angular velocity compared to controls, at less degree compared with PD. These alterations—typically associated with parkinsonism^8^—indicate a slowness of turning even in the prodromal phases of PD and even in the absence of subthreshold parkinsonism.

In TUG performed at fast speed, the differences of turning duration, and angular velocities between controls and PD were more evident compared to controls. Conversely, none of these parameters differentiated iRBD from controls – this is explained by a significantly higher percentage of variation change of duration between normal and fast speed in iRBD, compared to that of PD and of controls. We hypothesize that the lack of differences in the high-speed TUG between iRBD and controls could be due to a greater involvement of cortical circuits, which are proved to be hyperactivated in iRBDs during motor tasks^9^. Moreover, we hypothesize that subcortical networks responsible for usual mobility performance may be affected by the neurodegenerative process at an earlier time point compared with cortical networks responsible for challenging mobility performances in older adults^10,11^. When parkinsonism is present, conversely- the differences between patients and controls appeared to be even wider in challenging conditions compared to normal speed. This fits with several studies addressing gait and mobility changes in early PD^12^.

Inertial sensors are therefore promising tools that are able to detect subtle changes that are not captured by the evaluation of the time required to complete the task.

The study entails some limitations. First, although the sample size is large for these specific groups, it may still be too small to consider all confounding factors. However, the pattern of non-motor features in our iRBD cohort is comparable to previous cohorts with confirmed high conversion rates^5^, which argues for the relevance of the reported findings. An important strength of the study is the strict inclusion criteria for iRBD (PSG-confirmed, absence of subthreshold parkinsonism) and PD (drug-naïve and confirmed by dopaminergic imaging).

Second, the study design did not allow the calculation of the predictive value of digital parameters to conversion and need to be verified in on-going longitudinal multi-center studies. Third, we were not able to include an unsupervised assessment, as the features related to bradykinesia are expected to be more evident in the home-setting compared to supervised evaluation in the clinic^13^. Still, the data showed an important difference of turning time and speed even in the absence of changes in total TUG time in iRBD, thus confirming the potential usability of this measure as progression marker. One of the most important current limitations in prodromal PD research is indeed the lack of markers able to track subtle changes of disease progression at this stage. This issue is highly relevant for the research community, considering the upcoming availability of disease-modifying treatments.

In summary, this Mobile health technology-based study shows altered turning behavior in iRBD compared to controls, even in the absence of subthreshold parkinsonism and with a similar total TUG duration. Longitudinal studies are warranted to establish the sensitivity to change of turning alterations over time in prodromal PD phases. Understanding the underlying mechanisms of these mobility deficits could be pivotal to develop outcome parameters for pharmacological and non-pharmacological strategies in this early phase of alpha-synucleinopathies.

## Methods

### Participants and clinical assessment

The prospective cross-sectional study enrolled a total of 148 participants at the outpatient Movement Disorder clinic, Neurology Unit at the University of Brescia, Italy from April 2018 to November 2022 and at the Sleep disorder Centres of ASST Spedali Civili of Brescia and San Raffaele University, Milan. The research protocol was approved by the Ethics Committee of the Brescia Hospital, Brescia, Italy (DMA study, NP 1471). Written informed consent was obtained from all participants.

The study involved established iRBD individuals with a PSG-proven diagnosis and Drug-naïve PD patients^14^.

Established iRBD individuals were included based on the following criteria: (i) history of dream-enacting behavior; (ii) PSG proven REM sleep with sustained electromyographic (EMG) activity; (iii) absence of known pathological neurologic condition; (iv) lack of motor or cognitive complaints, (v) normal magnetic Resonance imaging (MRI) and (vi) lack of another sleep disorder, medical disorder or medication interfering with gait, or substance abuse^2^; (vii)-lack of abnormalities of gait at standard neurological examination.

Drug naïve patients with clinically evident PD symptoms^14^ were included. The following exclusion criteria were applied: (i) symptoms or features suggesting atypical parkinsonism; (ii) dementia^14,15^; (iii) other neurological disorders or medical conditions potentially associated with gait alterations; iv) abnormal MRI (v) need of walking aids; vi) normal nigrostriatal dopaminergic imaging; (vii) bipolar disorder, schizophrenia, history of drug or alcohol abuse or impulse control disorder.

Controls were recruited from patients’ families and from healthy volunteers.

Each participant underwent a standard medical and neurological examination, including the Movement Disorder Society- Unified Parkinson Disease Rating Scale (MDS-UPDRS)^16^, the Non-Motor Symptoms Scale for Parkinson’s Disease (NMSS)^17^, the Sniffing Sticks for the assessment of olfaction^18^, the Montreal Cognitive Assessment (MoCA) for the assessment of cognitive function^19^.

### Mobile health technology-instrumented Timed Up and Go assessment

All individuals were asked to perform the Timed Up and Go test (TUG). The TUG test was chosen because it embeds turning in a sequence of everyday movements, providing a more realistic patient’s ability to turn. The test was performed in clockwise and counterclockwise direction at normal and fast speed according to previous protocols^8,20^.

During the assessment, Rehagait® Inertial Measurement^21^ Unit with gyroscopes and accelerometers (Hasomed, Magdeburg, Germany) were used. The device was placed at the level of the fifth lumbar spine segment close to the center of mass. Signal raw data were downloaded to a computer, segmented into individual walking trials using time stamps, and analyzed by a bespoke MATLAB (R2021a) program. For the detection of turns and the extraction of turn parameters, we used a previously published algorithm developed by Pham et al.^22^. The development of the algorithm for turning detection and analysis was performed with MATLAB R2015b and consisted of three steps: 6DOF attitude estimation, turning detection, and turning analysis. The basic principle of the 6DOF attitude estimation is built on the relative orientation of the sensor with respect to the reference frame (global frame, G-frame), using a rotation matrix GSR (S stands for sensor frame, S-frame).

In the final step of the 6DOF attitude estimation, GS*R* was converted to Euler angles (roll–pitch–yaw) for the detection of turning, where roll represents the angle displacements around *X*, pitch around *Y*, and yaw around *Z*. Only yaw (i.e., *the angular displacement around the Z axis*) was considered for the next step.

Angular displacement around the Z axis was then plotted, where the start of a turn to the right was defined by a change from an increase to a decrease and the end by the change from a decrease to an increase. For the definition of a turn to the left, the situation was defined vice versa. The duration (horizontal component of the line) and magnitude (vertical component of the line) of each turn were determined between the start to end of the turn.

Turn direction was identified by integration of raw data from the gyroscope. A negative integration value was defined as a left turn and a positive integration as a right turn.

The turn metrics were duration, mean angular velocity and peak angular velocity (in degrees per second) of every turn^22^.

Only variables with a percentage of missing data lower than 5% were considered. Outliers were defined by a value higher or lower than 3 standard deviations of the disease-specific group and were excluded from the analyses.

The percentage of variation in fast speed in comparison to normal speed was calculated as follows for mean and peak angular velocities: % variation= fast velocity- normal velocity/normal velocity.

### Statistical analyses

Differences in demographic and clinical parameters between participants with iRBD, PD and controls were checked for their normality distribution with Shapiro–Wilk test and assessed with parametric ANOVA for continuous variables and chi-square test for categorical variables. Accordingly, either parametric or non-parametric univariate analysis were used for between-group analyses, adjusting for age and sex. Similar to Van Uem et al.^8^, mean values from the clockwise and counterclockwise turns were used. Between-group differences from the turning parameters were calculated using ANCOVA adjusting for age and sex. Bonferroni correction was applied to the post-hoc pairwise comparisons.

All analyses were 2-tailed, and *p* < 0.05 considered as statistically significant. For the analyses of digital parameters, a multiple comparison adjustment was adopted and statistical significance was set to *p* < 0.006. Statistical analyses were performed using IBM SPSS statistics version 26.

### Supplementary information

Supplementary Material

## Data Availability

The data that support the findings of this study are not openly available due to reasons of sensitivity and are available from the corresponding author upon completion of a specific request form (including data needed, objectives of the study/analyses and a specific data sharing agreement- provided by the corresponding author andrea.pilotto@unibs.it). Data are located in controlled access data storage at University of Brescia.
