# Supplementary material for: Turning alterations detected by mobile health technology in idiopathic REM sleep behavior disorder
Source: NPJ Parkinsons Dis. 2024 Mar 18;10:64. doi: 10.1038/s41531-024-00682-6 (PMC10948811; doi:10.1038/s41531-024-00682-6)
Supplement: Supplementary file 1 — Supplementary Material [file 41531_2024_682_MOESM1_ESM.pdf]

Supplementary Table 1. *Clockwise and counterclockwise parameters in HC, iRBD and PD*

	HC	iRBD	PD	P-value	HC-iRBD[c]	HC-PD[c]	iRBD-PD[c]
			TUG NORMAL SPEED LEFT				
TUG duration (s)	11.51±2.20	13.14±1.87	14.13±3.60	<0.001[b]	0.059	<0.001	0.542
Duration Of Turns (s)	2.26±0.43	2.75±0.46	2.93±0.65	<0.001[a]	0.021	<0.001	0.173
Mean Angular Velocity (°/s)	81±17	66±10	61±15	<0.001[a]	0.105	<0.001	0.024
Peak Angular Velocity (°/s)	191±35	156±27	134±30	<0.001[a]	0.006	<0.001	0.002
			TUG FAST SPEED LEFT				
TUG duration (s)	9.01±1.62	9.25±0.99	10.73±2.36	<0.001[b]	1.000	<0.001	0.007
Duration Of Turns (s)	1.86±0.34	2.2±0.49	2.43±0.49	<0.001[b]	0.347	<0.001	0.001
Mean Angular Velocity (°/s)	99±20	85±17	73±15	<0.001[a]	0.279	<0.001	0.001
Peak Angular Velocity (°/s)	236±42	206±44	172±38	<0.001[a]	0.223	<0.001	<0.001
			TUG NORMAL SPEED RIGHT				
TUG duration (s)	11.13±1.85	12.32±1.32	13.81±3.51	<0.001[b]	0.080	<0.001	0.298
Duration Of Turns (s)	2.33±0.43	2.75±0.46	2.93±0.65	<0.001[b]	0.214	<0.001	0.127
Mean Angular Velocity (°/s)	82±14	66±9	61±15	<0.001 [a]	0.009	<0.001	0.080
Peak Angular Velocity (°/s)	198±36	148±22	140±31	<0.001 [a]	<0.001	<0.001	0.064
			TUG FAST SPEED RIGHT				
TUG duration (s)	9.04±1.66	9.05±0.85	10.99±2.57	<0.001 [b]	1.000	<0.001	<0.001
Duration Of Turns (s)	1.91±0.36	2.17±0.44	2.51±0.55	<0.001[a]	0.939	<0.001	<0.001
Mean Angular Velocity (°/s)	98±19	86±16	71±17	<0.001 [a]	0.213	<0.001	<0.001
Peak Angular Velocity (°/s)	240±43	199±34	171±36	<0.001 [a]	0.006	<0.001	0.001

a. Parametric comparison corrected for age and sex

b. Non-parametric comparison corrected for age and sex

c. Pairwise with Bonferroni Correction

Supplementary Table 2. *Clockwise and counterclockwise parameters in PD with left and PD with right side most affected*

	PD Left side (41)	PD Right side (43)	P-value
Age [y]	68±8	67±8	0.488[a]
Sex M/F (%M)	24/17 (58)	25/18 (58)	0.971[b]
MDS-UPDRS-III (0-132) [pts]	15±9	16±9	0.428[a]
	TUG NORMAL SPEED LEFT		
TUG duration (s)	13.8±4.3	14.1±3.8	0.510[a]
Duration Of Turns (s)	2.9±0.7	3±0.7	0.522[a]
Mean Angular Velocity (°/s)	61.8±14.5	60.1±16.1	0.660[a]
Peak Angular Velocity (°/s)	134.1±30.4	136.8±35.4	0.792[a]
	TUG FAST SPEED LEFT		
TUG duration (s)	10.6±2.8	10.6±2.6	0.596[a]
Duration Of Turns (s)	2.4±0.6	2.5±0.5	0.616[a]
Mean Angular Velocity (°/s)	74.5±17	74.9±16.5	0.941[a]
Peak Angular Velocity (°/s)	173.3±42.1	172.9±38.2	0.892[a]
	TUG NORMAL SPEED RIGHT		
TUG duration (s)	13.6±4.1	13.7±3.7	0.473[a]
Duration Of Turns (s)	2.9±0.7	3.1±0.7	0.267[a]
Mean Angular Velocity (°/s)	62±15.5	60.1±15.1	0.517[a]
Peak Angular Velocity (°/s)	142.1±34.5	140.9±33	0.888[a]
	TUG FAST SPEED RIGHT		
TUG duration (s)	10.7±3.0	10.8±2.4	0.457[a]
Duration Of Turns (s)	2.4±0.6	2.6±0.6	0.226[a]
Mean Angular Velocity (°/s)	73.3±16.4	71.3±19.5	0.637[a]
Peak Angular Velocity (°/s)	171.3±36.7	170.3±41.9	0.677[a]

a. U-Mann Whitney
b. Chi-squared

Supplementary Table 3.

Literature review of motor progression studies in prodromal PD patients

Study	Participants	Motor assessment	Results
Beavan et al., 2015 (1)	30 homozygous GBA mutated, 28 heterozygote GBA mutated 26 controls	UPDRS-III	UPDRS-II higher in GBA mutation carriers
Avenali et al., 2019 (2)	31 homozygous GBA mutated, 16 heterozygote GBA mutated 16 controls	MDS-UPDRS III	MDS-UPDRS-II higher in GBA mutation carriers
Mirelman et Al, 2011 (3)	52 first degree relatives of PD patients mutated in LRRK2: 27 noncarriers of LRRK2 mutation and 25 carriers	An accelerometer quantified gait during usualwalking, fast-walking, and dual-tasking	stride time variability significantly increased especially under challenging conditions in mutation carriers
Mirelman et Al, 2016 (4)	122 LRRK2 mutation carriers, 67 PD, 186 HC	Different speed walking and dual task walking evaluated with synchronized 3-axis body-fixed sensors on the lower back and bilateral wrists	LRRK2 carriers and PD presented higher asymmetry and variability in comparison to controls in arm swing
Fereshtehnejad et al., 2019 (5)	152 iRBD<br>102 controls<br>69 PD	UPDRS III motor score<br>Facial expression<br>Speech and voice<br>Bradykinesia score<br>Tremor score<br>Rigidity score<br>PIGD score<br>Alternate tapping test<br>Purdue pegboard<br>Gait speed	Motor examination abnormalities began 5–7 years before phenoconversion, with the alternate tap test having the longest interval (8 years before phenoconversion). Among cardinal motor phenotypes, bradykinesia appeared first, 5–6 years prior to phenoconversion, followed by rigidity (Year –3) and tremor (Year –2).
Schalkamp et Al., 2023 (6)	Prospective population of 500.000 individuals, 1984 converted to PD	Accelerometry data	Reduction in acceleration before diagnosis unique to PD.
McDade et Al, 2013 (7)	42 probable RBD subjects using the Mayo Sleep Questionnaire and 492 controls	automated gait analysis system for gait velocity, cadence, and stride dynamics	Diagnosis of probable RBD associated with decreased velocity and cadence, significantly increased double limb support variability and greater stride time variability and swing time variability
Del Din et Al, 2019 (8)	696 healthy controls, 16 individual converted to PD	walk at different speeds under single and dual tasking, with a wearable device placed on the lower back	Higher step time variability and asymmetry of all gait characteristics associated with a shorter time to PD diagnosis
Martens et Al, 2019 (9)	24 iRBD, 14HC	walk at different speeds under single and dual tasking	Significant differences between the two groups in fast-speed walking and dual-task gait conditions.
Ma et al. 2021 (10)	31 iRBD, 20HC	walk at different speeds under single and dual tasking evaluated with wearable sensors	Decreased trunk motion and increased step time before turning may be possible prodromal symptoms of PD.

References:

1. Beavan M, McNeill A, Proukakis C, Hughes DA, Mehta A, Schapira AH. Evolution of prodromal clinical markers of Parkinson disease in a GBA mutation-positive cohort. JAMA Neurol. 2015;72(2):201-8.
2. Avenali M, Toffoli M, Mullin S, McNeil A, Hughes DA, Mehta A, et al. Evolution of prodromal parkinsonian features in a cohort of GBA mutation-positive individuals: a 6-year longitudinal study. J Neurol Neurosurg Psychiatry. 2019;90(10):1091-7.
3. Mirelman A, Gurevich T, Giladi N, Bar-Shira A, Orr-Urtreger A, Hausdorff JM. Gait alterations in healthy carriers of the LRRK2 G2019S mutation. Ann Neurol. 2011;69(1):193-7.
4. Mirelman A, Bernad-Elazari H, Thaler A, Giladi-Yacobi E, Gurevich T, Gana-Weisz M, et al. Arm swing as a potential new prodromal marker of Parkinson's disease. Mov Disord. 2016;31(10):1527-34.
5. Fereshtehnejad SM, Yao C, Pelletier A, Montplaisir JY, Gagnon JF, Postuma RB. Evolution of prodromal Parkinson's disease and dementia with Lewy bodies: a prospective study. Brain. 2019;142(7):2051-67.
6. Schalkamp AK, Peall KJ, Harrison NA, Sandor C. Wearable movement-tracking data identify Parkinson's disease years before clinical diagnosis. Nat Med. 2023;29(8):2048-56.
7. McDade EM, Boot BP, Christianson TJ, Pankratz VS, Boeve BF, Ferman TJ, et al. Subtle gait changes in patients with REM sleep behavior disorder. Mov Disord. 2013;28(13):1847-53.
8. Del Din S, Elshehabi M, Galna B, Hobert MA, Warmerdam E, Suenkel U, et al. Gait analysis with wearables predicts conversion to parkinson disease. Ann Neurol. 2019;86(3):357-67.
9. Ehgoetz Martens KA, Matar E, Hall JM, Phillips J, Szeto JYY, Gouelle A, et al. Subtle gait and balance impairments occur in idiopathic rapid eye movement sleep behavior disorder. Mov Disord. 2019;34(9):1374-80.
10. Ma L, Liu SY, Cen SS, Li Y, Zhang H, Han C, et al. Detection of Motor Dysfunction With Wearable Sensors in Patients With Idiopathic Rapid Eye Movement Disorder. Front Bioeng Biotechnol. 2021;9:627481.
